# Unintended consequences of healthcare reform in South Korea: evidence from a regression discontinuity in time design

**DOI:** 10.1186/s12961-023-00993-9

**Published:** 2023-06-22

**Authors:** Moon Joon Kim

**Affiliations:** Department of Economics, George Mason University Korea, Songdomunhwa-ro 119-4, Yeonsu-gu, Incheon, 21985 South Korea

**Keywords:** Healthcare utilization, Copayment, Regression discontinuity in time, Adverse selection, Moral hazard

## Abstract

**Background:**

To address concerns over the financial stability of South Korea’s National Health Insurance (NHI) programme, the government transitioned from an outpatient copayment system to a coinsurance system in 2007. This policy aimed to reduce healthcare overutilization by increasing patients’ financial responsibility for outpatient services.

**Methods:**

Using comprehensive data on NHI beneficiaries, this study employs a regression discontinuity in time (RDiT) design to assess the policy’s impact on outpatient healthcare utilization and expenditures. We focus on changes in overall outpatient visits, average healthcare cost per visit and total outpatient healthcare expenditures.

**Results:**

Our findings indicate that the transition from outpatient copayment to coinsurance led to a substantial increase in outpatient healthcare utilization (up to 90%) while decreasing medical expenditures per visit by 23%. The policy shift incentivized beneficiaries to seek more medical treatments during the grace period and enroll in supplemental private health insurance, which provided access to additional medical services at lower marginal costs.

**Conclusions:**

The policy change and the emergence of supplemental private insurance contributed to moral hazard and adverse selection issues, culminating in South Korea becoming the country with the highest per capita utilization of outpatient health services worldwide since 2012. This study underscores the importance of carefully considering the unintended consequences of policy interventions in the healthcare sector.

## Background

South Korea consistently ranks at the top among Organisation for Economic Co-operation and Development (OECD) countries in terms of per capita outpatient healthcare utilization, raising concerns about the nation’s healthcare quality [33]. In 2017, the annual number of doctor consultations per capita in South Korea reached 16.6, an impressive six visits more than second-tier countries such as Hungary and Slovakia. This increase in outpatient visits puts considerable strain on the existing healthcare system, resulting in longer patient wait times, shorter consultations and overworked doctors who cannot provide their full attention to each patient. Consequently, South Korea’s healthcare quality indicator has experienced a downward trend since 2008, with scores dropping from 115 in 2008 to 94.5 in 2015 [33].

The Korean government is grappling with a rapidly widening gap in per capita annual outpatient visits compared with second-tier countries. According to OECD Health Statistics [33], Japan held the top spot for per capita outpatient visits until 2009, while South Korea ranked third in 2002. However, South Korea’s sharp increase in the annual average number of outpatients per capita propelled it to second place in 2005 and the top spot in 2010. Excluding 2011, when Japan reclaimed the lead, South Korea has consistently ranked first among OECD countries since 2012. More concerning is the growing disparity between South Korea and second-tier countries, which has expanded dramatically over the years: 1.4 (versus Japan in 2012), 1.8 (versus Japan in 2013), 3.6 (versus Japan in 2014), 3.2 (versus Japan in 2015), 4 (versus Japan in 2016) and 5.7 (versus Hungary and Slovakia in 2017). This striking trend underscores the urgent need for a comprehensive examination of the nation’s healthcare policies and practices to safeguard the well-being of its citizens.

In the early 2000s, the Korean government confronted a critical issue: the copayment programme was suspected of contributing to excessive, low-cost medical use, jeopardizing the financial stability of the National Health Insurance (NHI) programme. In response, a groundbreaking medical amendment was passed in 2007, transitioning from the copayment programme to a coinsurance system with the goal of increasing patients’ financial share of healthcare costs and curbing the overuse of medical services. This pivotal change marked a crucial turning point in the nation’s strategy for addressing growing concerns surrounding healthcare utilization.

### Korean healthcare system and outpatient copayment programme

The National Health Insurance Service (NHIS) in South Korea is a single, compulsory national insurance system under the Ministry of Health and Welfare. Covering 97.1% of the population as of December 2022, the NHIS ensures access to healthcare for all residents, including overseas Koreans and foreign nationals. The remaining 2.9% of low-income households receive support through the Medical Aid programme.^1^

Since its inception in 1963, the National Health Insurance Corporation (NHIC) has required a coinsurance policy for inpatient care (20%) and doctor visits (30%). However, concerns emerged over excessive healthcare usage due to the low patient sharing of coinsurance costs. To address this, the NHIC implemented a copayment policy in 1987 to increase patient sharing by up to 50%. This policy effectively reduced healthcare utilization, as the average medical cost led to a patient sharing percentage higher than the original 30% coinsurance rate [26].

However, the copayment policy did not adequately adjust for economic development and inflation, causing patient shares to decrease over time. By 2001, patient shares had fallen to 22%, and the NHI fixed the copayment at 3000 South Korean won(KRW) for medical bills equal to or below 15 000 KRW, stating that the copayment rate would remain unchanged in the future. Consequently, outpatient utilization rates continued to increase, and South Korea ranked third among OECD countries for per capita outpatient healthcare services usage in 2007. This led to criticism of the copayment policy for driving up total medical costs due to the overuse of medical services.

In response, the government passed medical reforms in June 2007, abolishing the copayment policy and reinstating the coinsurance system to curb excessive use of medical services and promote the financial stability of the NHI. Since August 2007, beneficiaries have been responsible for at least 30% of the total outpatient care costs, while seniors aged 65 years and older can continue to use the existing copayment programme, and children under 6 years old have a reduced copayment rate.

### Supplemental private health insurance

Many countries have introduced private health insurance to complement their national health insurance (NHI) systems. For instance, in Germany, the introduction of private insurance has led to an increased choice of healthcare providers and improved financial stability of the healthcare system [5]. Similarly, in Australia, the introduction of private health insurance incentives has been associated with increased uptake of private health insurance and reduced waiting times in public hospitals [9].

The United States Medigap, a form of private supplemental insurance, shares similarities with private insurance in South Korea. Medigap policies have been found to increase healthcare utilization among Medicare beneficiaries, particularly among those with chronic conditions [14]. However, some studies argue that Medigap contributes to increased Medicare spending due to moral hazard and adverse selection [12, 23].

Following the government’s decision to allow private insurance into the market in October 2003, the landscape of health insurance in South Korea experienced a significant transformation [22]. The goal of this decision was to alleviate the financial strain on the NHIS due to excessive medical service usage and to lessen the economic burden on patients suffering from conditions not covered by the NHIS. Consequently, the South Korean health insurance market saw the emergence of a wide range of supplementary private health insurance products designed to complement the NHI coverage. These supplementary private health insurance plans typically cover the cost of copayments or coinsurance, providing policyholders with additional financial protection in the event of illness or injury. Moreover, some of these plans also cover services and treatments not included in the NHI, such as dental care, vision care and alternative medicine.

The rise of private insurance in South Korea has led to several notable outcomes. First, it has broadened access to healthcare services for many individuals, as they can now afford treatments and services that were previously out of reach due to cost constraints [22]. This has contributed to the overall improvement in the quality of care received by patients and, in some cases, has resulted in better health outcomes. Second, the availability of private insurance has provided households with a greater sense of financial security. By offering coverage for costs not covered by the NHI, these plans help policyholders avoid catastrophic out-of-pocket expenses, which can have devastating consequences for families in terms of debt and financial stress [22]. However, the proliferation of supplementary private health insurance has also raised concerns about potential negative effects on the healthcare system, such as increased healthcare utilization [43], moral hazard and adverse selection.

Drawing from the existing literature, this study aims to enhance our understanding of the implications of transitioning from outpatient copayment to coinsurance, as well as the role of supplemental private insurance in South Korea. Utilizing rigorous empirical analysis, this research investigates the impact of the shift from outpatient copayment to coinsurance policy on healthcare utilization, costs and access to care, ultimately contributing to future discussions surrounding the optimal design of healthcare financing systems.

## Methods

### Data

This study utilizes national hospitalization data for NHI beneficiaries in South Korea from 2002 to 2015. The NHI, a compulsory single health insurer, covers 97.1% of the total population as of 2022, while the remaining 2.9% are covered by the Medical Aid programme.^2^ The NHI tracks all medical records for both inpatients and outpatients under its coverage. To enable public disclosure, the NHI randomly selects 1 million beneficiaries each year from those who visited hospitals at least once during the year,^3^ and includes all their hospitalization records for that year.

The primary focus of this study is national outpatient healthcare utilization. This approach is informed by several factors. First, the copayment policy applied solely to outpatient medical costs equal to or less than 15 000 KRW (US$ 13.64).^4^ Moreover, the change in healthcare policy affected outpatient healthcare services for all diseases covered by the NHI. Estimating the average treatment effect on a daily basis is challenging; therefore, the data are aggregated by month to examine changes in hospitalization rates. Lastly, this paper considers outpatient visits across all age groups. Although the copayment abolishment excluded children (0–5 years) and seniors (65+ years), this study aims to investigate the effects of supplemental private health insurance, which emerged due to the transition from outpatient copayment to coinsurance. The analysis also considers vulnerable population groups, such as children and the elderly, who are more likely to have supplemental private health insurance supported by their guardians.

The dataset used in this analysis spans all cities and provinces in Korea over a 14 year period from 2002 to 2015. To investigate changes in outpatient utilization, we exclude inpatient hospitalization data and observations where the sum of patient sharing and NHI sharing does not equal the total healthcare cost due to data collection errors. The total number of outpatient observations amounts to 105 million. As estimating the average treatment effect using daily hospital outpatient visit data is challenging, the data are aggregated by month to concentrate on the monthly variation in hospitalization. Consequently, we construct panel data using monthly average hospitalization data from 16 cities and provinces.^5^

Table 1 presents the summary statistics for the key variables employed in this study. *Monthly hospital visit* is approximately 40 000; however, this figure is not particularly informative as it is derived from sample data. Instead, we use a natural logarithm transformation to determine the percentage of changes in healthcare utilization. *Age* represents the monthly average age of outpatients. The NHIS provides age group data, and we use the mean number of each age group for each beneficiary. For instance, if a beneficiary belongs to the age group between 20 and 24 years old, 22 years is used for the subscriber’s age. *Sex* is a dummy variable that equals 1 if the beneficiary is female and 0 otherwise. An average sex ratio of 0.58 implies that women visit physicians more frequently than men. The medical cost per visit amounts to 19 839 KRW (US$ 18) per patient, with the patient paying 6011 KRW (US $5.47), and the remaining 13 804 KRW (US$ 13) covered by the NHI. Patients share approximately 30% of the per-visit medical cost, while the NHI contributes the remaining 70%.

**Table 1:** Summary statistics

	*N*	Mean	SD	Min	Max
Monthly hospital visit	2688	39 193	43 926	2796	228 693
Age	2688	42.86 years	3.75 years	31.64 years	55.49 years
Sex	2688	0.58	0.01	0.53	0.61
Spending per-visit	2688	19 839 KRW	3260 KRW	14 821 KRW	29 815 KRW
		(US$ 18)	(US$ 3)	(US$ 14)	(US$ 27)
– Patient-sharing	2688	6011 KRW	888 KRW	4176 KRW	8993 KRW
		(US$6)	(US$1)	(US$4)	(US$8)
– NHI-sharing	2688	13 804 KRW	2454 KRW	10 108 KRW	21 267 KRW
		(US$ 13)	(US$ 2)	(US$ 9)	(US$ 19)

This table displays the number of observations (*N*), as well as the mean, standard deviation (SD), minimum (min) and maximum (mad) values for the key variables used in this paper, which are derived from the monthly aggregated numbers for each city/province. Sex is the ratio of female patients to male patients

### Empirical strategy

To evaluate the causal effect of the new policy or its changes, randomized controlled trials (RCTs) or other quasi-experimental methodologies are typically preferred for establishing treatment and control groups and estimating the differences in outcomes. However, in this study, the change in the national programme affected all NHI beneficiaries, constituting approximately 97% of the Korean population. As a result, it is difficult to estimate the causal effect of healthcare reform due to the lack of a clean control group.

To address this challenge, we employ a regression discontinuity in time (RDiT) approach to analyse changes in outpatient healthcare utilization before and after the transition from outpatient copayment to coinsurance. First, we implement a parametric RDiT regression to assess the treatment effects of eliminating the copayment programme on healthcare utilization and expenditures. Second, we estimate the average treatment effect using a local linear regression at the cutoff *τ*, following Calonico et al. [6] and Roh [39]. Then, we repeat the RDiT regression using various alternatives, such as including covariates and different bandwidths above and below the cutoff.

Applying the RDiT design to the nationwide healthcare policy change offers several benefits. First, this policy change affected everyone living in Korea, thereby preventing self-selection problems. Second, the policy could not have been anticipated, and therefore, its implementation and timing can be considered random. People had little incentive to manipulate hospital visits before the amendment was enacted. Although the amendment was expected to pass, people were aware that there would be a grace period during which they could still benefit from the copayment programme.

### Regression discontinuity in time to estimate LATE

For identification purposes, we assume that outpatient hospital utilization and expenditures changed immediately upon the passage of the medical amendment in June 2007, rather than in August 2007 when the policy was actually implemented. The rationale is that NHI beneficiaries responded as soon as the health insurance reform was confirmed. The running variable is time *t*, and treatment status is defined as:

1 $$D_{{ct}} = \left\{ {\begin{array}{*{20}c} 1 & {{\text{if}}\,{\mkern 1mu} t \ge \tau } \\ 0 & {{\text{otherwise}}} \\ \end{array} } \right.$$

where *D*_*ct*_ is a dummy variable for the transition from outpatient copayment to coinsurance, *τ* represents the cutoff (June 2007) and *c* and *t* denote city and time, respectively.

The baseline parametric RDiT specification to estimate the causal effects of healthcare reform on healthcare utilization and expenditure is given by:

2 $$Yct = \alpha + \beta {\text{1}} \cdot Dct + \beta {\text{2}}\cdot\psi (Zct){\text{ }} + \beta {\text{3}} \cdot Xct + \eta c + \epsilon ct$$

where *β1*, the coefficient of interest, estimates the causal effect of transitioning from outpatient copayment to coinsurance. The *X* variable includes control variables such as average age and sex ratio. The city fixed effect, *ηc*, is incorporated to control for unobserved heterogeneity between cities and provinces. *ψ(Z*_*ct*_*)* represents a continuous function of the running variable *t* with a second-degree polynomial.

Cluster-robust standard errors are utilized at the city/province level to account for within-cluster serial autocorrelation. Clustering considers the within-cluster correlation of standard errors, avoiding excessively small standard errors that would result in narrower confidence intervals, larger *t*-statistics, and lower *P*-values [7].

Furthermore, a nonparametric RDiT model is employed to estimate the discontinuity in the conditional expectation *E*[*Y*_*ct*_|*Z*_*ct*_] at the cutoff. A local polynomial model, using only observations within a given bandwidth around the cutoff, is preferred over the global approach. Including observations far from the threshold may lead to bias in estimating the local average treatment effect (LATE) [17].

For the polynomial order, a first-order polynomial is used, as recommended by Gelman and Imbens [17]. High-order polynomials can result in significant approximation errors, such as noisy estimates, sensitivity to the polynomial degree and poor confidence interval coverage. Skovron and Titiunik [42] also argue that a lower-order polynomial is preferred when employing an optimal bandwidth selector, as it allows for flexible bandwidth size adjustment for better approximation within a given polynomial order. The preferred local linear RDiT estimator in this study is defined as follows:

3 $${\widehat{\beta }}_{RD}={\widehat{\alpha }}_{+}+{\widehat{\alpha }}_{-}$$

where ${\widehat{\alpha }}_{+}$ and ${\widehat{\alpha }}_{-}$ are defined through

4 $${\widehat{\phi }}_{Y}=\underset{ {\widehat{\alpha }}_{+}, {\widehat{\alpha }}_{-} , \widehat{\lambda }}{\mathrm{argmin}}\sum {\{{Y}_{ct}-{D}_{ct}\left({\alpha }_{+}+{\lambda }_{+}{Z}_{ct}\right)-\left(1-{D}_{ct}\right)\left({\alpha }_{-}+{\lambda }_{-}{Z}_{ct}\right)\}}^{2}\cdot K(\frac{{Z}_{ct}}{b})$$

where ${\widehat{\phi }}_{Y}=\left[{\alpha }_{+},{\alpha }_{-}, {\lambda }_{+}, {\lambda }_{-}\right]$, *K*(·) is a kernel function, and *b* is its bandwidth.

The local linear RDiT estimator ${\widehat{\beta }}_{RD}$ is estimated by the vertical distance between the intercepts of the weighted linear regression [${\widehat{\alpha }}_{+}, {\widehat{\alpha }}_{-}$] applied separately to the left and right of the cutoff [39]. The weighting is determined by the kernel function *K*(*Z*_*ct*_*/b*) in Eq. 4; specifically, a triangular kernel function is utilized, where *K*(*u*) = 1|*u*|≤ 1·(1 − |*u*|). This function assigns zero weight to all observations outside the bandwidth interval [*x*_0_ − *b, x*_0_ + *b*] and positive linear down-weighting to the observations within the interval. This approach results in a point estimator with optimal variance and bias properties [6, 39, 41].

For the bandwidth, a data-driven mean squared error (MSE) optimal bandwidth selection, proposed by Calonico et al. [6], is applied. This updated version of the optimal bandwidth selector offers different MSE optimal bandwidths above and below the threshold while employing the same kernel function for units in each bandwidth.

## Results

**Fig. 1:**
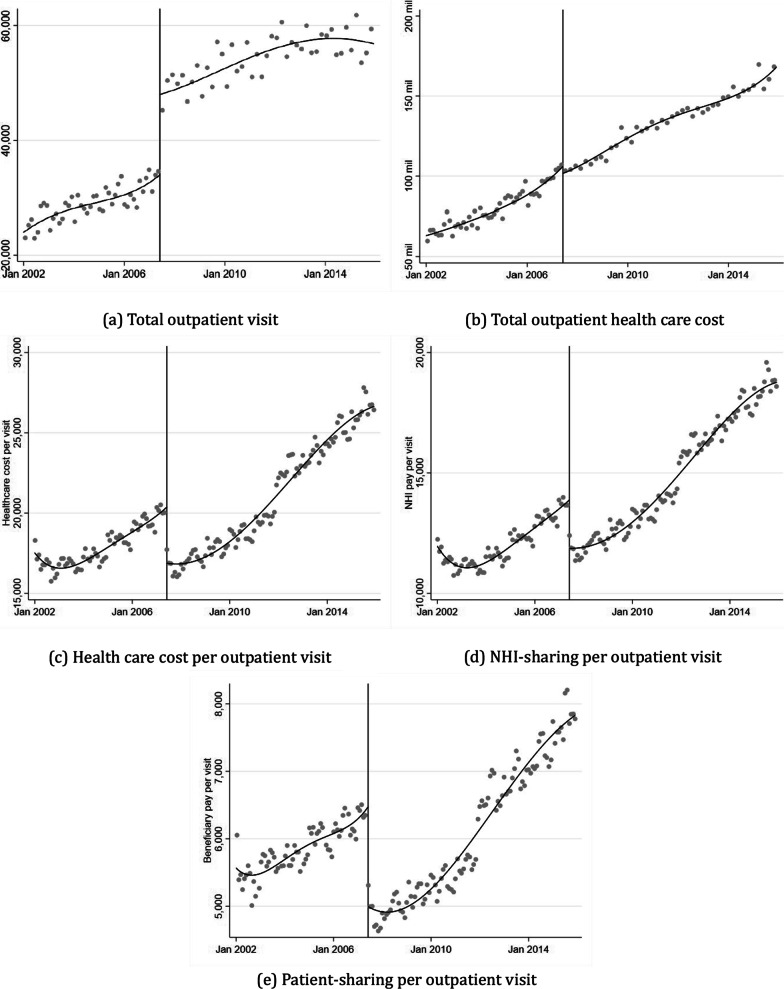
Discontinuity in healthcare utilization

In addition to regression analyses, graphical evidence offers valuable insights for estimating programme effects using an RDiT design. As such, this paper first examines the graphical evidence and then discusses the regression results. Figure 1 displays the monthly average outpatient hospital visits and their related costs from January 2002–December 2015. A local linear regression is fitted to estimate $\widehat{\alpha }$ for each side of the cutoff month, June 2007, when the amendment was passed. On the left-hand side of the cutoff, prior to June 2007, an increasing trend in healthcare utilization is observed. The Korean government, concerned that this trend could threaten the sustainability of the NHI in the future, decided to terminate the copayment programme, which was suspected to be the primary cause of healthcare overuse. If the transition from outpatient copayment to coinsurance had a significant effect, outpatient hospital visits at the cutoff point would be expected to experience a sharp decrease.

Contrary to expectations, Fig. 1a demonstrates that the number of outpatient visits increased sharply as soon as the healthcare policy amendment was passed in June 2007, providing evidence that the transition from outpatient copayment to coinsurance has significantly impacted the growth of outpatient healthcare utilization. Outpatient visits evidently increase at the cutoff, and visually, the monthly outpatient healthcare use grows by approximately 100%. In contrast, Fig. 1b presents the total healthcare spending during the study period. Despite the discontinuous increase in healthcare use at the threshold, there is no graphical evidence of significant changes in total healthcare expenditures, suggesting a trade-off between the number of hospitalizations and the cost of each medical care use.

Figure 1c illustrates the changes in medical expenses per visit. Per-visit healthcare costs experience a sharp decrease in the cutoff month, explaining why total medical expenditure remains unchanged in the cutoff month, despite the significant increase in healthcare utilization. The increase in hospital visits with more minor symptoms can lead to a decrease in average healthcare costs per visit; thus, we find no graphical evidence of changes in overall healthcare spending.

The regression results, however, show some divergence from the graphical findings. Columns 1–3 in Table 2 present the results from the fixed effects model with different manually applied bandwidths. The first column displays the results for the entire study period, while the outcomes shown in the second and third columns are based on 6-month and 3-month intervals before and after the cutoff point, respectively. As the bandwidth narrows, the effect’s magnitude decreases from around 60–30%, yet the estimates remain statistically significant. Columns 4 and 5 provide estimates based on the parametric and local linear models using Eqs. 2 and 4, respectively. Although the estimates of the parametric models are statistically significant and their magnitudes are also comparable to the estimates of the fixed effects model in Columns 1–3, the estimates of the local linear model are positive but not statistically significant.

**Table 2:** Regression discontinuity regression results

	1	2	3	4	5
	All	[-6m, +6m]	[-3m, +3m]	Parametric	Local linear
Panel A: total outpatient visit					
Copayment abolition	0.471***	0.404***	0.247***	0.383***	0.322
	(0.013)	(0.014)	(0.010)	(0.020)	(0.258)
	[60.16%]	[49.78%]	[28.12%]	[46.67%]	[37.99%]
*R*^2^	0.955	0.975	0.973	0.964	
Panel B: total healthcare cost					
Copayment abolition	0.215***	0.031	−0.103***	0.021	−0.045
	(0.013)	(0.018)	(0.016)	(0.021)	(0.266)
	[23.99%]	[3.15%]	[−9.79%]	[2.12%]	[−4.40%]
*R*^2^	0.892	0.770	0.650	0.964	
Panel C: healthcare cost per-visit					
Copayment abolition	−0.255***	−0.373***	−0.350***	−0.363***	−0.264***
	(0.015)	(0.012)	(0.013)	(0.008)	(0.018)
	[−22.51%]	[−31.13%]	[−29.53%]	[−30.44%]	[−23.20%]
*R*^2^	0.653	0.984	0.984	0.964	
Panel C-1: healthcare cost per visit (NHI-sharing)					
Copayment abolition	−0.233***	−0.364***	−0.357***	−0.352***	−0.273***
	(0.015)	(0.013)	(0.015)	(0.009)	(0.018)
	[−20.78%]	[−30.51%]	[−30.02%]	[−29.67%]	[−23.89%]
*R*^2^	0.567	0.982	0.981	0.964	
Panel C-2: healthcare cost per visit (patient-sharing)					
Copayment abolition	−0.306***	−0.392***	−0.332***	−0.386***	−0.246***
	(0.014)	(0.011)	(0.011)	(0.010)	(0.021)
	[−26.36%]	[−32.43%]	[−28.25%]	[−32.02%]	[−21.81%]
*R*^2^	0.808	0.986	0.985	0.964	
Observations	2688	192	96	2688	2688

Columns 1–3 show the regression results from a fixed effects model. The regression results from a parametric model and a local linear model are presented in Columns 4–5. The average age variable is included as a covariate. *** Significant at the 1% level, ** Significant at the 5% level, * Significant at the 10% level.

To explore the discrepancy between the graphical evidence and the insignificant regression results, we revisit the investigation of the cause behind the increase in hospital utilization. As previously mentioned, the transition from outpatient copayment to coinsurance led healthcare subscribers to become more concerned about higher hospital expenses, which motivated using private health insurance as a safeguard against this expense increase. If private insurance subscribers were responsible for the rise in outpatient services due to moral hazard, which implies the excessive use of medical services with a marginal cost approaching zero, there would be some changes in the characteristics of outpatient users that contribute to an endogenous effect on outpatient healthcare utilization.

**Fig. 2:**
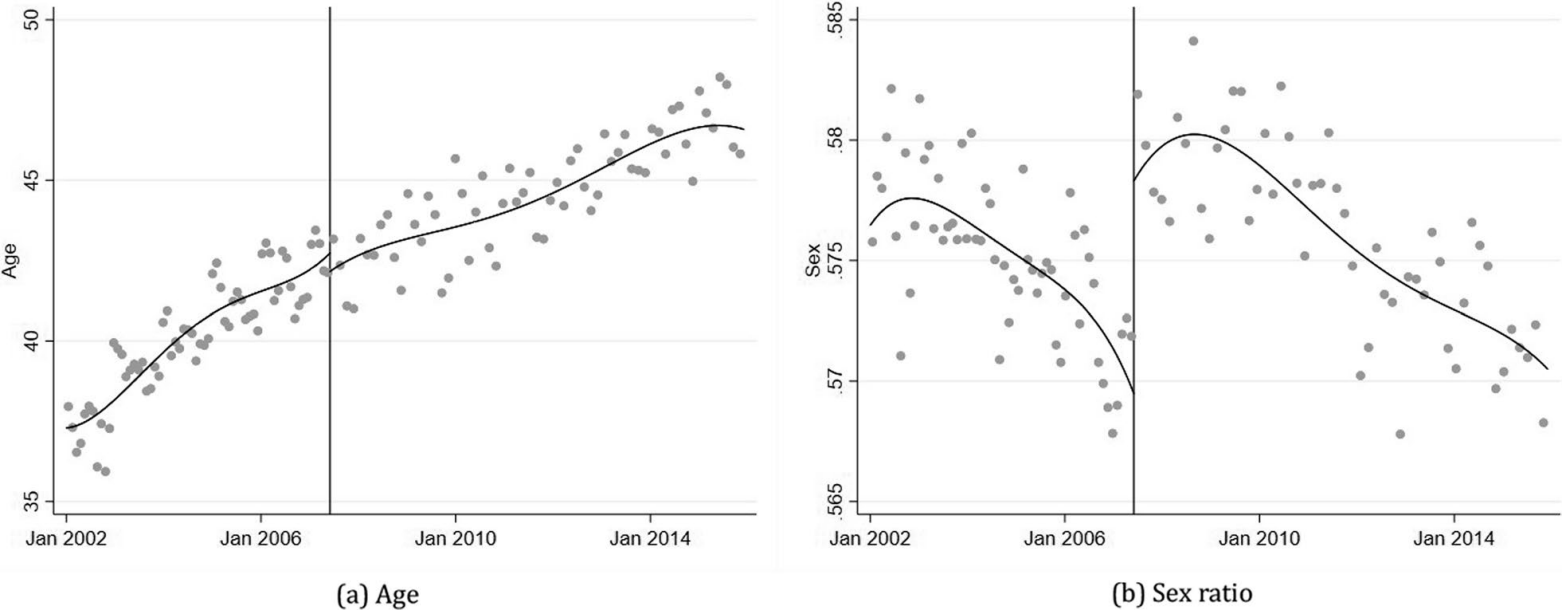
Discontinuity in covariates

Two variables can be employed to examine changes in the characteristics of outpatient medical users using a given dataset: *Age* and *Sex ratio*. Figure 2 presents the monthly average age and sex ratio of outpatient medical users throughout the study period. As illustrated in Fig. 2a, the average age of outpatients follows an upward curve due to ageing, but there is no significant difference in the increase or decrease at the threshold. In contrast, in the graph of average sex ratio in Fig. 2b, a very clear increase is visible based on the cutoff point. Given that the sex ratio is composed of indicator variables with 0 for males and 1 for females, it can be inferred that the outpatient utilization of women has increased rapidly after the medical law amendment passed. This result can be interpreted in various ways, but the most plausible interpretation concerns the sex differences in the utilization of healthcare services.

Over the past several decades, research has demonstrated that women tend to use more healthcare services than men [2, 11, 13, 45]. This difference is primarily attributed to biological and behavioural factors [36], socio-economic and socio-demographic status differences [10] and variations in the perceptions of illness [19, 38]. Noh et al. [32] corroborate that, in Korea, women utilize more healthcare services than men.

Drawing a definitive conclusion is challenging due to the limited information on private health insurance subscribers. Nevertheless, based on the presented results, the transition from outpatient copayment to coinsurance prompted people to seek private health insurance as a safeguard against rising medical costs. South Korean women, who are more likely to utilize healthcare services, appear to be particularly proactive in obtaining private health insurance. Moreover, the marginal cost of using additional medical services after enrolling in private insurance dropped nearly to zero, leading to a substantial increase in medical service utilization due to the moral hazard presented by private insurance subscribers. Sohn and Jung [43] also argue that individuals with private health insurance plans tend to use outpatient visits more frequently than those who only have NHI.

If the rapid increase in the use of medical services can be attributed to private insurance, and if the participants in this private insurance are primarily women, a fuzzy RDiT design can be employed. This design assumes that healthcare subscribers are partially enrolled in private insurance, and the assignment to treatment (enrolling in private insurance) depends on the sex ratio. Column 1 in Panel A of Table 3 presents the regression results using fuzzy RDiT, revealing that the average number of monthly outpatient users increased by approximately 90% due to the elimination of the copayment programme. However, even after controlling for endogeneity, the estimates remain positive but statistically insignificant. The total healthcare cost exhibits no significant change, while the per-visit healthcare cost is significantly reduced by about 23%. Consequently, similar to the previous findings, the substantial decrease in per-visit healthcare costs makes it challenging to identify a significant change in total healthcare costs, despite the increased utilization of healthcare services, as depicted in Panel B.

**Table 3:** Local linear RD regression results

	1	2	3
	Total	Single	Multiple
Panel A: total outpatient visit			
Copayment abolition	0.633	0.744**	−0.816**
	(0.479)	(0.309)	(0.253)
	[88.30%]	[110.47%]	[−55.77%]
RD type	Fuzzy	Fuzzy	Fuzzy
Panel B: total healthcare costs			
Copayment abolition	−0.045	0.475*	−0.625**
	(0.266)	(0.261)	(0.289)
	[−4.40%]	[60.80%]	[−46.47%]
RD type	Sharp	Sharp	Sharp
Panel C: healthcare costs per visit			
Copayment abolition	−0.264***	−0.099***	0.364***
	(0.018)	(0.011)	(0.035)
	[−23.20%]	[−9.43%]	[43.91%]
RD type	Sharp	Sharp	Sharp
Observations	2688	2688	2688

Column 1 includes all outpatient visits. Columns 2 and 3 only include one-time visits and multiple visits, respectively. Covariate variables are included in the regression. A cluster–adjusted standard error is used to account for the within-cluster correlation. We use a polynomial of order one and a triangular kernel function. A data-driven mean squared error optimal bandwidth selection is applied. *** Significant at the 1% level, ** Significant at the 5% level, * Significant at the 10% level.

The observed decline in healthcare costs per visit raises important questions. According to Bundorf et al. [4], the cost per visit in healthcare may be correlated with the severity of a patient’s ailment. Outpatient fees generally encompass the quality of care, consultation duration and supplementary medical services such as injections and bandages [3]. As a result, when the illness is relatively mild or extra medical care is unnecessary, the cost of each medical visit will be lower [37]. Thus, the decline in per-visit medical expenses could suggest that patients visiting healthcare facilities have less severe illnesses [29]. This occurrence is frequently linked to the moral hazard of private insurance policyholders seeking to maximize their use of hospital services for minor ailments due to the decreased marginal cost of hospital visits [35].

Changes in illness severity can be identified by examining the number of times healthcare services are used for the same condition. If the illness is mild, doctors are unlikely to recommend more than one visit for medical treatment for the same illness. Since Korea is among the countries with the highest number of medical services per capita in the world, doctors do not typically recommend additional medical services for relatively minor illnesses. However, if the condition is severe or chronic, patients may use outpatient services more than once for the same condition. Consequently, this study distinguishes between two cases: (1) a single visit to outpatient services, and (2) multiple visits for the same condition.

**Fig. 3:**
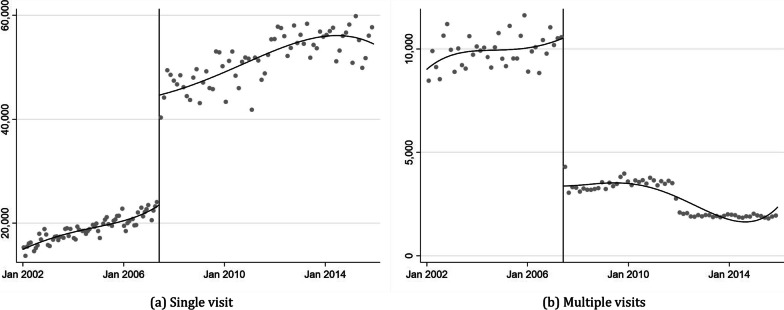
Outpatient visit comparison between single visit and multiple visits

Figure 3 displays the average number of visits per month for patients using outpatient services for the same disorder once and for those using it more than once. As illustrated in (a), there is a significant increase in the number of monthly outpatient patients who make a single visit for a condition after the medical law amendment. Conversely, the number of patients using medical services more than once due to the same condition decreases sharply (2 (b)). Columns 2 and 3 in Panel A of Table 3 indicate that the use of a single outpatient service for patients increases by approximately 110%, while multiple visits show a reduction of approximately 56%; both estimates are statistically significant. These results suggest that the increased use of healthcare services for mild symptoms leads to a rise in total healthcare utilization. Simultaneously, the decrease in the number of multiple uses of healthcare services stems from an adverse selection problem, wherein relatively healthy individuals use more healthcare services.

The sudden increase in outpatient utilization resulting from the transition from outpatient copayment to coinsurance can be attributed to the new coinsurance programme and private insurance subscribers. As the increase in medical expenses was anticipated to exacerbate the economic burden on national health insurers, people began actively using private insurance as a defense mechanism. Women, who are more sensitive to changes in market prices, predominantly participate in private insurance. As a result, privately insured individuals find it easier to access healthcare services for even mild illnesses due to the lower marginal cost. The increased use of one-time hospital services serves as evidence of moral hazard, but it may also indicate adverse selection, which refers to a situation where relatively healthy people disproportionately use more healthcare services, leading to imbalances in the risk pool while the number of multi-service users decreases.

### Robustness checks and validity tests

In this section, we conduct robustness and validity checks to address potential confounding factors in the RDiT estimation. First, we investigate placebo effects by examining various subgroups that the healthcare reform should not impact. Next, we address specific concerns associated with the RDiT approach.

#### Placebo tests

The healthcare reform on patient cost-sharing was only applied to the population aged between 6 and 65 years. The elderly group aged 65 years and above remained eligible for the copayment programme, while the children aged below 6 years paid 70% of the cost borne by adults. Consequently, outpatient utilization by children and the elderly should be independent and should not be impacted by the transition from outpatient copayment to coinsurance. To test for placebo effects, we replicate the RDiT regressions by focusing on two age groups: children aged 0–4 years and seniors aged 65 years and above. If the discontinuity in outpatient visits is solely driven by an increase in patient burden in the population aged below 65 years, no discontinuity should exist in outpatient utilization among the elderly aged 65 years and above at the cutoff. However, if private health insurance proliferated due to the transition from outpatient copayment to coinsurance, the healthcare utilization of other age groups is expected to be affected.

Table 4 presents the regression results of the placebo tests. Column 1 displays the overall healthcare utilization rate by age group. Interestingly, a significant increase in healthcare utilization was observed in the age groups 5 years and under and 65 years and older, representing individuals unaffected by the transition from outpatient copayment to coinsurance. However, we do not find any significant changes in healthcare utilization in the target age group between 5 and 64 years. This finding offers two important implications. First, the policy intervention aimed at increasing the economic burden of patients through the transition from outpatient copayment to coinsurance was unsuccessful. Second, this intervention incentivized NHI beneficiaries to take a greater interest in private insurance covering all cost-sharing expenses, resulting in moral hazard and adverse selection problems among vulnerable populations.

**Table 4:** Placebo tests

	1	2	3
	Total	Single	Multiple
Panel A: age 0–4 years			
Copayment abolition			
	0.488***	0.985***	−1.637***
	(0.230)	(0.242)	(0.254)
	[62.91%]	[167.78%]	[−80.54%]
RD type	Sharp	Sharp	Sharp
Panel B: age 5–64 years			
Copayment abolition			
	0.272	0.681**	−0.927***
	(0.265)	(0.293)	(0.269)
	[31.26%]	[97.59%]	[−60.43%]
RD type	Sharp	Fuzzy	Sharp
Panel C: age 65+ years			
Copayment abolition			
	0.437*	0.491*	−0.437***
	(0.241)	(0.261)	(0.157)
	[54.81%]	[63.39%]	[−35.40%]
RD type	Sharp	Fuzzy	Fuzzy
Observations	2688	2688	2688

Column 1 includes all outpatient visits. Columns 2 and 3 only include one-time visits and multiple visits, respectively. Covariate variables are included in the regression. A cluster–adjusted standard error is used to account for within-cluster correlation. We use a polynomial of order one and a triangular kernel function. A data-driven mean squared error optimal bandwidth selection is applied. *** Significant at the 1% level, ** Significant at the 5% level, * Significant at the 10% level.

#### Spillover effects

The healthcare reform concerning copayment was limited to outpatients who did not need to stay overnight in the hospital, so this policy change was not expected to affect inpatient health utilization. However, if the change in cost-sharing encouraged NHI beneficiaries to obtain supplemental private health insurance covering patient-sharing for inpatient and outpatient services, it would be reasonable to suspect spillover effects on inpatient healthcare utilization.

**Fig. 4:**
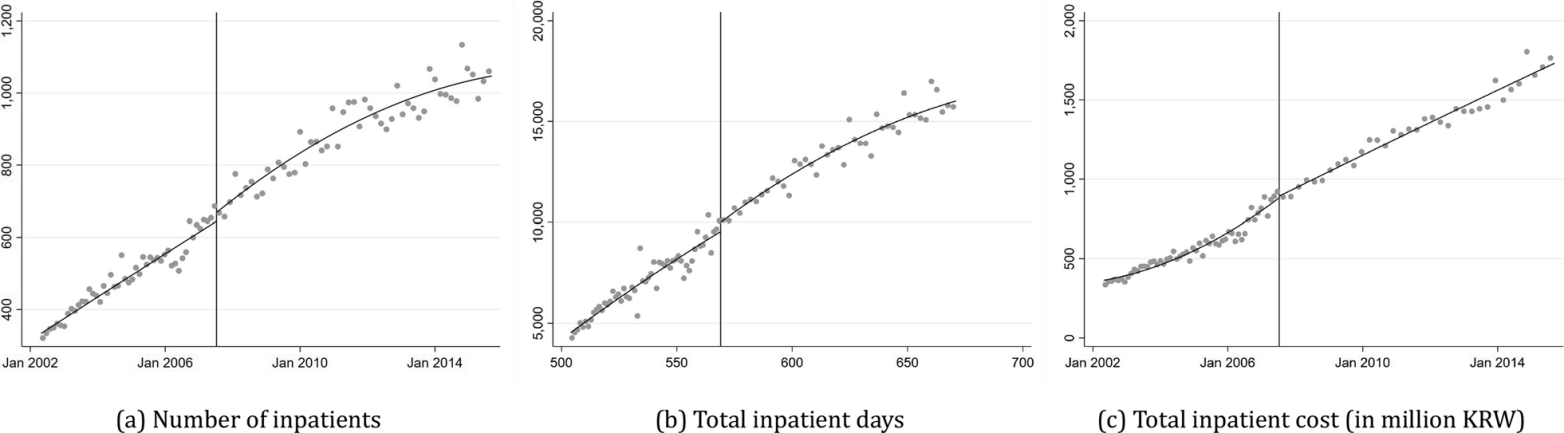
Spillover effect – inpatient

Figure 4a illustrates the trend in the number of hospitalized patients during the study period. The vertical line in the middle represents the cutoff, June 2007, when the medical amendment was passed. No significant discontinuity is observed at the threshold, implying that additional private health insurance did not substantially impact inpatient healthcare access. This might be due to the referral system for inpatient admission. Generally, unlike outpatients, who do not require a referral from a doctor for consultation, patients must display significant symptoms necessitating continuous monitoring over a period to be hospitalized.

Moreover, no evidence of moral hazard, such as inpatients staying in the hospital longer than expected or requiring more expensive and/or unnecessary treatments during hospitalization, is found. Figures 4b, c depict the total number of hospitalization days and the cost of inpatients, respectively. No significant discontinuities or jumps at the threshold are observed. The consistently increasing slope of the number of inpatients, total inpatient days, and total inpatient costs before and after the threshold suggests that supplemental private health insurance is not associated with inpatient care. This could be because all decisions regarding hospitalization, medications and medical technology are made by physicians, primarily due to asymmetric information.

### Validity tests of RDiT

While it is assumed that the treatment assignment is as good as random near the cutoff, many cross-sectional RD studies have necessitated various validity tests to examine potential biases, such as the data sorting effect or anticipating effect. However, these standard validity tests are irrelevant to RD studies using time as a running variable. For instance, the density test of sorting behaviour proposed by McCrary [30] cannot be applied, because the density of the time running variable is uniform and, as a result, has no discontinuity in the density of the running variable at the cutoff.

Hausman and Rapson [18] discussed the differences between cross-sectional RD and RDiT designs. Due to the unique characteristics of RD in time, which rely on time-series variation for identification, the authors suggest that researchers using RDiT should review a checklist. In the context of this paper, we carefully examine each concern on the list as follows.

#### Unobservables correlated with time

In a cross-sectional RD model, including a covariate may be helpful in reducing noise and increasing the accuracy of the estimates, but it is optional and not required [28]. However, in the RDiT setting, incorporating a covariate in the regression model is strongly recommended due to the potential correlation with the discontinuity of the running variable. In this study, the age of the beneficiaries, which is closely related to health status, is included as a control variable. The age variable helps control for the increase in outpatient healthcare usage patterns during the study period. Additionally, the fixed effects model and clustered standard errors account for heterogeneity and the time trends of the unobservables in each cluster, which might be correlated with the discontinuity around the cutoff.

#### Time-varying treatment effects

The treatment effect in RDiT specifications might not be constant and could vary over time; however, current RDiT settings do not support testing for time-varying effects. Previous studies have used difference-in-differences or qualitatively estimated short- and long-run impacts to discuss time-varying treatment effects in an RDiT. In this paper, we assume that the treatment effect is smooth and constant throughout the post-period for two reasons. First, as shown in Fig. 1a, outpatient hospital utilization consistently increased after the initial increase at the cutoff. Second, this consistent and parallel increase in healthcare utilization can be explained by the effects of the grace period, supplemental private insurance and coinsurance programme, as discussed in the results section.

#### Autoregressive properties

Since RDiT relies on time-series variation, two concerns regarding autoregression are proposed: serial correlation in residuals and lagged dependent variables. First, the serial dependence in the residuals is controlled for by clustering the standard errors in both the parametric and local linear specifications, allowing for dependency within the clusters while maintaining independence between the clusters. Second, outpatient healthcare services are typically used every 2 or 3 days after an initial visit and generally do not last longer than 3 or 4 weeks. Therefore, monthly aggregated hospitalization data are used in this study to control for the lagged effects on the dependent variable.

#### Selection and strategic behaviour

In many cross-sectional RD models, data manipulation near the cutoff by sorting behaviour or anticipation effects is examined using the McCrary [30] test. However, because the running variable is time in RDiT specifications, testing the discontinuity of the running variable is not possible. Instead, we investigate any events that might impact the discontinuity of the outcome variable near the threshold.

**Fig. 5:**
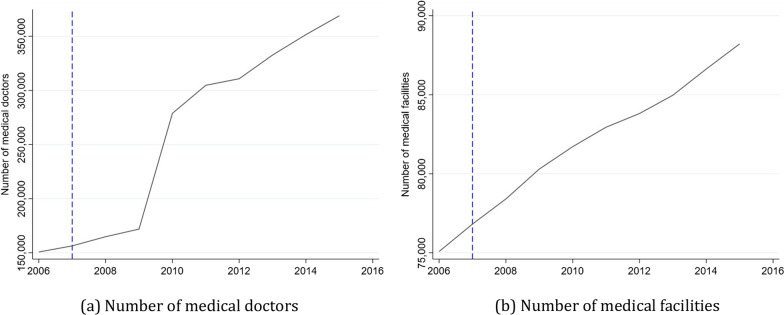
Health care supply trends between 2006 and 2015

**Fig. 6:**
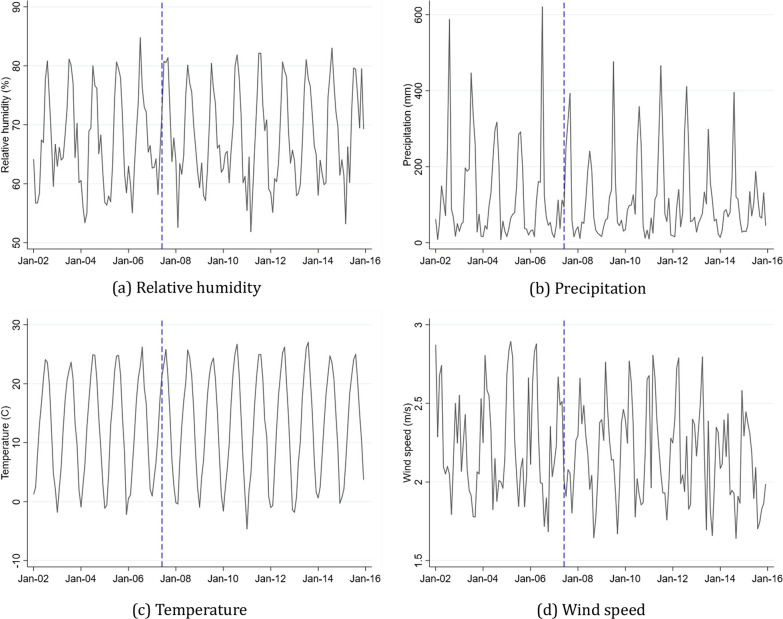
Weather trends between 2002 and 2015

Fig. 5 displays the trends of healthcare services supply from 2006 to 2015, with the blue dashed line representing the year 2007. Around the time of interest, we do not find any discontinuous changes in either the number of medical doctors (A.1.a) or the number of medical facilities (A.1.b). Furthermore, Fig. 6 shows the monthly trends of meteorological conditions during the study period, with the blue dashed line indicating June 2007. Again, no significant changes in relative humidity (A.2.a), precipitation (A.2.b), temperature (A.2.c) or wind speed (A.2.d) occurred around June 2007. Additionally, no other changes in healthcare policy or epidemic disease events were identified at the time of interest, June 2007.

## Discussion

Why did this policy change, which aimed to increase patients’ economic burden, lead to an increase in healthcare services utilization? First, there was a 2-month grace period between when the amendment was passed (June 2007) and when it was implemented (August 2007), during which NHI subscribers increased their demand for medical care services before the cost of healthcare rose (Einav et al. 2013). Although NHI subscribers may have experienced only minor symptoms, they sought to visit their doctors more frequently during the grace period to avoid higher costs in the future.

Second, the patient-sharing burden decreased after the policy implementation due to the emergence of supplemental private insurance and the new coinsurance programme. As supplemental private insurance reimburses the patient’s share of copayment or coinsurance, the increased cost-sharing for NHI subscribers led to a rapid increase in private insurance enrolment. Consequently, the marginal cost for medical services approached almost zero, making outpatient access to medical services significantly more affordable [35]. Sohn and Jung [43] suggest that the introduction of private insurance in South Korea has led to increased outpatient visit frequency for individuals with private health insurance plans. Furthermore, outpatients with private insurance paid a monthly premium of 8000 KRW (approximately US$ 7) for a 40-year-old man in 2007, which may have incentivized the overuse of medical services to justify the monthly premium payments.

Additionally, under the new coinsurance programme, patient sharing per visit became even less expensive for some minor symptoms. Therefore, reduced patient sharing due to private insurance and coinsurance made medical care services more affordable, particularly for mild symptom treatments such as colds and flu [1].

This paper contributes to the literature in three dimensions. First, it uses a quasi-experimental design to estimate how people respond to changes in healthcare cost-sharing plans, allowing us to derive a causal relationship between healthcare costs and utilization [16].

Second, the findings suggest that increased patient cost-sharing does not necessarily reduce healthcare utilization, in contrast to the existing literature [15, 31, 40]. This result implies that demand for healthcare may still respond to price changes. Still, people seek alternative options to reduce increased cost-sharing and may potentially use more healthcare services if an alternative financial service ultimately reduces the patient share [21, 41]..

Lastly, this paper examines the factors that encouraged enrolment in supplemental private insurance and the subsequent increase in low-value outpatient healthcare utilization. Research has shown that supplemental private health insurance may increase the use of healthcare services due to moral hazard and adverse selection problems [12, 23, 25]. However, there is little discussion about the policy interventions that cause existing health insurance beneficiaries to opt for supplemental private health insurance.

This study has crucial policy implications. First, the use of supplemental private health insurance should be restricted to mid- or high-value healthcare services, and copayment ought to be reintroduced for low-value healthcare services. The impact of copayment on healthcare utilization has been extensively documented [8, 20, 44], and copayment programmes can still effectively increase patients’ financial burden for low-value medical services. Consequently, this study advocates for the reintroduction of copayments in the South Korean healthcare market, albeit at a slightly higher rate and with greater flexibility. This would allow for periodic adjustments in accordance with the inflation rate [27].

Second, the shift from outpatient copayment to coinsurance inadvertently promotes private insurance enrolment, which in turn causes marginal healthcare costs to approach zero, leading to excessive healthcare utilization by private insurance subscribers. This finding highlights the importance of carefully managing changes to existing policies or introducing new policies. Policy-makers should consider all potential impacts before implementing changes; otherwise, unintended consequences may arise from the interaction effects between related policies.

## Conclusions

In 2007, South Korea transitioned from an outpatient copayment programme to a coinsurance system, aiming to reduce the financial burden on the NHI. Additionally, supplemental private health insurance emerged as a means to alleviate the financial burden on NHI beneficiaries. While both changes achieved some of their intended goals, they also brought about unintended consequences that require further examination.

On the positive side, the shift to coinsurance contributed to improved access to healthcare services and reduced out-of-pocket expenses for patients, particularly among vulnerable groups. This policy change led to increased healthcare utilization, especially among the elderly population. Moreover, supplemental private health insurance helped to further alleviate the financial burden on NHI beneficiaries.

However, these changes also introduced some negative outcomes. The transition to coinsurance may have inadvertently encouraged moral hazard by making patients more likely to seek unnecessary medical care. The increased demand for healthcare services also led to higher overall healthcare costs. As for supplemental private health insurance, it potentially contributed to the overutilization of healthcare services by lowering the marginal cost of care for subscribers.

In conclusion, while the shift to coinsurance and the introduction of supplemental private health insurance achieved some positive outcomes in reducing the financial burden on the NHI and its beneficiaries, they also generated unintended consequences that must be carefully considered and addressed in future policy-making.

Despite the robustness checks and sensitivity analyses conducted in this paper, three caveats must be mentioned. First, there is a lack of information about supplemental private health insurance beneficiaries and changes in their enrolment rates near the cutoff. This limitation is primarily due to the inaccessibility of data on private insurance enrolment as a result of confidentiality concerns. The unavailability of such data poses a challenge in examining the direct impact of the policy change on the enrolment behaviour in private insurance.

Furthermore, while this paper assumes that the increase in healthcare utilization was primarily attributable to supplemental private insurance beneficiaries, it is worth noting that outpatient visits may also have increased due to lower patient cost-sharing under the new coinsurance programme. However, this factor cannot be directly measured within the scope of this study setting.

Lastly, as mentioned in the validation section, the conventional RD design validity tests are inapplicable in this setting because of the time-based running variable. This paper employs the guidelines proposed by Hausman and Rapson [18] to address the validity concerns of RDiT designs. However, additional research is necessary to validate regression discontinuity designs that incorporate a time running variable.

## Data Availability

The data generated and analysed in this study are not publicly available due to Korea National Health Insurance Service regulations but are available from the author upon reasonable request.

## Appendix

See Figs. 5 and 6

## Abbreviations

KRW: South Korean won
LATE: Local average treatment effect
MSE: Mean squared error
NHI: National Health Insurance
NHIC: National Health Insurance Corporation
RCT: Randomized controlled trial
RD: Regression discontinuity
RDiT: Regression discontinuity in time

## Footnotes

1 https://www.nhis.or.kr (in Korean).

2 The Medical Aid programme in South Korea shares similarities with the Medicaid programme in the United States, as both initiatives aim to provide support for low-income households that struggle to afford healthcare expenses.

3 According to the OECD Health Data (2007), the annual per capita outpatient utilization rate in South Korea is 11.8. This indicates that using sample data based on beneficiaries who access outpatient healthcare services at least once a year should not pose any significant issues.

4 The average exchange rate for the study period (2002–2015) is approximately 1100 KRW per US$. This exchange rate is used throughout the study.

5 Sejong, established as a separate city in 2012, was originally part of Chungnam Province. For the sake of simplicity in this analysis, Sejong is included within Chungnam Province.
